# The Use of Diets to Improve the Quality of Life of Women With Breast Cancer

**DOI:** 10.7759/cureus.57718

**Published:** 2024-04-06

**Authors:** Fernanda C Poscai Ribeiro, Isadora Damasceno Queiroz, Fernando Ari Fernandes Alves, Samira El Maerrawi Tebecherane Haddad, Marcelo G Perseguino

**Affiliations:** 1 Department of Internal Medicine, University of Western São Paulo, Guarujá, BRA; 2 Department of Gynecology, University of Western São Paulo, Guarujá, BRA; 3 Department of Public Health, University of Ribeirão Preto, Guarujá, BRA

**Keywords:** eortc qlq-c30, breast neoplasm, quality of life (qol), diet, breast cancer

## Abstract

Introduction: There has been an increase in the incidence of breast cancer cases in the last decade, and despite the treatment increasing the chances of survival, it reduces the quality of life. In this context, diets could decrease the adverse effects of treatment and improve quality of life.

Methodology: A form with the European Organization for the Research and Treatment of Cancer Quality of Life Questionnaire, which contains specific scores for physical, cognitive, emotional, symptomatic, and functional performance, was made available in a Facebook support group. Afterward, the data were analyzed using linear regression and a t-test of independent samples using Jamovi version 2.3.24 (retrieved from https://www.jamovi.org).

Results: There was a low number of participants who followed the ketogenic diet or intermittent fasting. In general, adherence to the diets was good. In the t-test, diets showed improvement in physical performance. Linear regression correlated treatment with chemotherapy, metastases, and bad diet adherence with worse symptomatic scores.

Conclusion: There is evidence that diets can improve the symptoms of these patients; however, there is no consensus about which diet produces the best effect, requiring further studies on this subject.

## Introduction

According to the Brazilian National Cancer Institute of the Ministry of Health, it is projected that among a female population numbering approximately 93 million, there will be an estimated 48,930 cases of breast cancer. Breast cancer has witnessed a substantial upswing in both its incidence and crude mortality rates over the recent decades [1].

While therapeutic interventions have notably enhanced survival rates among breast cancer patients, they are not without repercussions, as they inflict harm upon healthy tissues and give rise to pronounced side effects, encompassing emotional distress, myelosuppression, fatigue, vomiting, and diarrhea. In this context, dietary modifications hold the potential to ameliorate the quality of life for individuals with breast cancer [2].

Fasting and caloric restriction exert influences on metabolism, potentially augmenting the morning cortisol levels and thereby bestowing favorable modulatory effects on cardiopulmonary fitness, body composition, fatigue, weakness, and overall quality of life. Furthermore, caloric restriction is posited to diminish the carcinogenic and metastatic propensities of cancer stem cells [3].

Additionally, specific diets, such as the ketogenic diet, involve the replacement of nearly all plant-derived carbohydrates, excluding non-starchy varieties, with reduced to moderate quantities of protein and elevated levels of monounsaturated and polyunsaturated fats. The justification for its employment in cancer therapy is substantiated by its potential to curtail carbohydrate uptake, potentially culminating in the apoptosis of malignant cells, while concurrently elevating the availability of ketone bodies for energy production within normal cells [4].

Recent studies have probed into the prospect of caloric restrictions, exemplified by intermittent fasting, as adjunctive therapies to heighten the efficacy of conventional cancer treatments like chemotherapy, radiation therapy, and emerging immunotherapies. Nevertheless, there remains a paucity of documented human investigations elucidating the tolerance, adherence, or efficacy of dietary interventions aimed at enhancing the quality of life in women afflicted with breast cancer [3-5].

## Materials and methods

The data were collected via a Google Forms (Google LLC, Mountain View, California, USA) questionnaire, which was made available within the Brazilian Facebook private group “Supere o Câncer de Mama” (link: https://www.facebook.com/groups/2866504250251277/?ref=share&mibextid=NSMWBT). In the selection of our research participants, a pivotal factor influencing our decision was the acquisition of appropriate authorization from the page owner. This step was deemed necessary to align with the ethical standards mandated by the research committee, ensuring adherence to regulatory protocols. Subsequently, upon securing the requisite authorization, our research endeavor garnered approval from the Research Ethics Committee of the São Paulo Western University (CEP-UNOESTE; approval number: 56738122.0.0000.55151).

**Figure 1:**
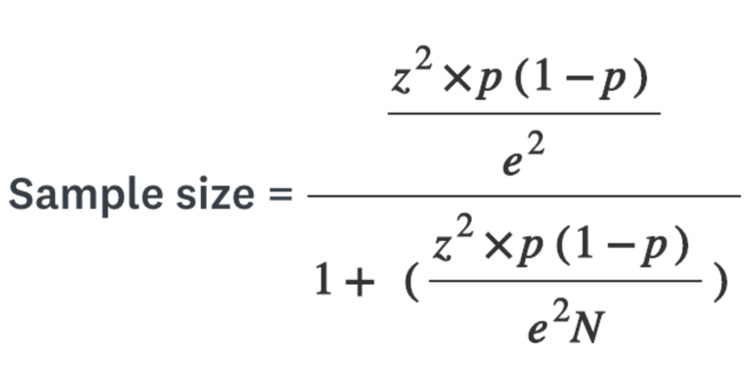
Sample size formula N: population size, e: margin of error, z: z-score

The data collection process commenced in June 2023 and concluded in August 2023. Initially, the group comprised 1214 members, increasing to 1398 by the end of the data collection period. Sample size calculations indicated that, with a population of this size, a confidence level of 95%, and a margin of error of 5%, the required sample size at the study’s outset was 292, which was subsequently adjusted to 302 by the study’s conclusion. The formula utilized for this sample size calculation is available in Figure 1.

The questionnaire comprises several key inquiries, including the patient’s dietary habits (none, intermittent fasting, ketogenic diet, another restrictive diet recommended by a healthcare professional that does not fit the criteria of ketogenic diet or intermittent fasting), duration of dietary adherence, presence of metastasis, molecular classification, treatment details, self-assessed adherence level to the diet (categorized as low, moderate, or high), and the patient’s perception of improvement in comparison to their previous state. Additionally, the questionnaire includes items from the Questionnaire for Quality of Life Assessment in Cancer Patients version 3.0 (EORTC QLQ-C30), which aims to assess the patient’s quality of life and was previously validated in Brazilian patients [6].

The EORTC QLQ-C30 questionnaire gauges various functional dimensions, such as physical, functional, emotional, cognitive, symptom-related, and social aspects scores. Comprising 30 items, this questionnaire is applicable to all cancer patients undergoing treatment.

Each question in the questionnaire is assigned a score, with higher scores indicating a poorer state. For instance, in response to the question, “Did pain interfere with your daily activities?”, the available responses are no (1), slightly (2), moderately (3), and significantly (4). Notably, the last two questions differ from this pattern, where higher scores signify better health and quality of life, ranging from 1 (excellent) to 7 (poor). To maintain consistency with the scoring trend, the Google Forms questionnaire equates “excellent” to a score of 1 and “terrible” to a score of 7.

Subsequently, we collected the EORTC QLQ-C30 scores from all participants and conducted an independent sample t-test to assess score differences (total, physical, functional, cognitive, emotional, social, and symptomatic) between groups that followed a specific diet regimen and those that did not. Moreover, a linear regression analysis was performed with the scores as the dependent variable and factors such as the presence of metastasis, treatment modalities (chemotherapy, radiotherapy, surgical removal, and immunotherapy), adherence to the diet, and classification as independent variables. Numeric variables were log-transformed to achieve a normal distribution, a prerequisite for linear regression. The statistical analysis was carried out using Jamovi version 2.3.24. The predetermined significance level for conducting analyses was set at p<0.05.

## Results

There were 303 responses recorded out of 1398, five of which were excluded due to missing information (age) and inconsistency. (They marked both the options that they performed diets and did not perform them simultaneously.) Therefore, only 298 were considered valid. Out of this total, 96 were on diets, and 202 were not. A detailed description of the sample is illustrated in Table 1.

**Table 1:** Descriptive information Luminal A: Tumors are ER (estrogen receptor) and PR (progesterone receptor) positive but HER2 (human epidermal growth factor receptor 2) negative. HER2Neu positive: It is ER and PR negative but HER2 positive. Luminal B: It is ER positive, PR negative, and HER2 positive. Triple-negative: Tumors are negative for ER, PR, and HER2.

Variables	Group that followed diets (n=96)	Group that did not follow diets (n=202)
Age in years		
Mean (SD)	44.73 (11.75)	44.63 (13)
Classification		
Luminal A, n (%)	18 (18.75)	27 (13.37)
Luminal B, n (%)	21 (21.87)	51 (25.25)
Unknown, n (%)	14 (14.58)	34 (16.83)
HER2Neu positive, n (%)	25 (26.04)	51 (25.25)
Triple-negative, n (%)	18 (18.75)	39 (19.30)
Treatment		
Chemotherapy, n (%)	51 (53.12)	117 (57.92)
Radiotherapy, n (%)	31 (32.3)	76 (37.62)
Immunotherapy, n (%)	20 (20.83)	37 (18.32)
Surgical removal, n (%)	38 (39.6)	92 (45.54)
Others, n (%)	29 (30.21)	34 (16.83)
Metastasis		
No, n (%)	69 (71.87)	151 (74.75)
Yes, n (%)	17 (17.71)	51 (25.24)

Regarding dietary information, 57 individuals (representing 59.37%) reported being on diets for more than three months, eight (8.33%) for approximately three months, 12 (12.5%) for around two months, seven (7.29%) for approximately one month, and 12 (12.5%) for less than one month. In terms of adherence to their respective diets, 44 participants (45.83%) classified their adherence as good, 46 (48.96%) as average, and only 10 (10.41%) classified it as poor. Notably, 72 patients (75%) reported feeling better after commencing their diets, while 23 patients claimed to feel neither better nor worse.

Regarding the three patients following the ketogenic diet, all of them adhered to it for more than three months, with two demonstrating good adherence and one classifying their adherence as poor. Among the seven patients following intermittent fasting, one rated their adherence as poor, three as average, and the remaining as good.

In the t-test results, the difference between the means of the groups that adhered to a diet and those that did not were mostly not significant across most categories, except for the physical score, which exhibited a slight advantage for the group adhering to a diet (Table 2).

**Table 2:** t-test results

					Confidence interval 95%	
	Statistic	p	Mean difference	Standard error of the difference	Inferior limit	Superior limit
Physical score	2.247	0.025	0.04794	0.0213	0.00594	0.0899
Total score	0.877	0.381	0.01425	0.0163	-0.01774	0.0462
Emotional score	0.568	0.570	0.01218	0.0214	-0.03001	0.0544
Functional score	0.795	0.427	0.01752	0.0220	-0.02587	0.0609
Social score	0.339	0.734	0.00863	0.0254	-0.04139	0.0587
Symptomatic score	0.894	0.372	0.01639	0.0183	-0.01969	0.0525
Cognitive score	-1.978	0.051	-0.04893	0.0247	-0.09760	-2.49e−4

In the context of linear regression, the collinearity statistics indicate that the independent variables do not exhibit autocorrelation, thereby affirming the validity of linear regression. However, according to the results of the Shapiro-Wilk test, normal characteristics were observed solely in the symptom-related score variable measured by EORTC QLQ-C30. Consequently, only the symptomatic score was employed as the dependent variable in the linear correlation analysis.

In the conducted linear regression analysis, the p-value obtained from the F test signifies that at least one of the variables is associated with the outcome. Specifically, in this instance, the identified variables linked to higher symptomatic scores are the presence of metastasis, chemotherapy, and adherence (Table 3-4).

**Table 3:** Model adjustment measures of linear regression R: the correlation coefficient, R²: the coefficient of determination, adjusted R²: a version of R² that has been adjusted for the number of predictors in the model, F: the F-statistic, df1: degrees of freedom for the model, df2: degrees of freedom for the residuals

				Global model test			
Model	R	R²	Adjusted R²	F	df1	df2	p
1	0.329	0.109	0.0476	1.78	19	278	0.025

**Table 4:** Linear regression coefficient models of total score

Predictor	Estimates	Standard error	t	p
Interceptᵃ	1.65321	0.1279	12.927	
Metastasis				
Yes - No	0.05485	0.0187	2.936	0.004
Chemotherapy				
Yes - No	0.03499	0.0175	1.997	0.047
Surgical removal				
Yes - No	-0.01014	0.0189	-0.535	0.593
Immunotherapy				
Yes - No	0.00235	0.0199	0.118	0.906
Radiotherapy				
Yes - No	0.02561	0.0204	1.253	0.211
Adherence to diets				
Bad - No diet	-0.05231	0.1423	-0.368	0.713
Medium - No diet	-0.12560	0.1425	-0.881	0.379
Good - No diet	-0.15134	0.1445	-1.047	0.296
Diet follow-up time				
>3 months - No diet	0.10349	0.1441	0.718	0.473
<1 month - No diet	0.16700	0.1466	1.139	0.256
Approximately 1 month - No diet	0.10386	0.1297	0.801	0.424
Approximately 2 months - No diet	0.11191	0.1469	0.762	0.447
Approximately 3 months - No diet	0.17809	0.1494	1.192	0.234
Classification				
HER2 superexpression - Luminal A	0.12189	0.1295	0.941	0.347
Unknown - Luminal A	0.10232	0.1299	0.788	0.432
Luminal B - Luminal A	0.10404	0.1294	0.804	0.422
Triple-negative - Luminal A	0.10261	0.1300	0.790	0.430

Regarding linear regressions, the only statistically significant finding was related to the symptom-related score. The Durbin-Watson test for autocorrelation shows a Durbin-Watson statistic of 1.91, a value close to 2, indicating no autocorrelation. The Shapiro-Wilk test applied to residuals demonstrated p=0.076, confirming a normal distribution. The other regressions were not performed as they did not meet the criteria for normal distribution (Shapiro-Wilk: p<0.05); therefore, the calculation could not be conducted. Also, the variance inflation factor (VIF) was lower than 4 and does not warrant further investigation (Table 5).

**Table 5:** Collinearity statistics VIF: variance inflation factor, tolerance: the reciprocal of VIF, where values less than 0.1 indicate higher collinearity

	VIF	Tolerance
Metastasis	1.06	0.945
Chemotherapy	1.17	0.853
Surgical removal	1.27	0.787
Immunotherapy	1.04	0.958
Radiotherapy	1.33	0.753
Adherence	2.19	0.456
Follow-up	1.60	0.624
Classification	1.04	0.963

## Discussion

Out of the 298 cases examined, only three were following a ketogenic diet, indicating a low rate of adherence to this dietary regimen. One potential explanation for this limited adoption of the ketogenic diet is its highly restrictive nature. Additionally, concerns among physicians regarding the significant increase in LDL levels associated with the ketogenic diet may contribute to their reluctance to recommend it [7-8].

Also, only seven patients adhered to intermittent fasting. Alongside the restrictive nature of this diet, the phenomenon of craving-induced overcompensation during limited mealtimes may lead to negligible or no calorie restriction. This factor likely accounts for the scarcity of patients choosing this dietary approach [9].

In summary, restrictive diets had fewer participants. Another possibility to consider is that patients may have received recommendations for ketogenic or intermittent fasting diets from nutritionists or other professionals, but due to a lack of familiarity with the correct terminology, some patients may have selected the “other” option even after reading brief descriptions of each diet in the questionnaire.

Regarding improvements observed, 75% of participants reported feeling better after initiating their respective diets. While this information is subjective, it’s important to note that emotions and sensations represent immediate physiological responses that inform the individual’s perception of a stimulus or situation [10]. It’s worth mentioning that even healthy individuals can experience worsened well-being when adopting a new diet, which can negatively impact adherence. Thus, this data holds promise [11].

Regarding adherence, 93.75% of patients classified their adherence as good or moderate. This suggests that patients with a heightened awareness of the severity of their condition tend to be more diligent in following their therapeutic regimen [12]. Furthermore, in linear regression analysis, poor adherence was associated with worsening symptoms. Other studies have shown that adhering to healthy diets can enhance overall quality of life, reduce symptoms, and even lower the risk of recurrence. Conversely, high adherence to unhealthy or Western dietary patterns has been linked to increased overall mortality [13-15]. Additionally, patients who adhered to their prescribed diets showed a slight improvement in physical function, as indicated by t-tests. Prior evidence suggests that dietary interventions, particularly in conjunction with physical activity, can enhance physical function during treatment. In patients with advanced cancer, maintaining physical function becomes crucial, as immobility is linked to an increased risk of sarcopenia [16-18].

During linear regression analysis, other components of the EORTC QLQ30 did not exhibit significant variations based on dietary patterns overall. However, further investigation is warranted for subgroups of breast cancer survivors. Previous studies have shown that participants receiving dietary interventions experienced significant improvements in quality-of-life measures [19-21].

Furthermore, the linear regression analysis demonstrates that the presence of metastasis and chemotherapy treatment have a detrimental impact on quality of life. The cytotoxic effects of chemotherapy predominantly affect rapidly dividing cells, such as those in hematopoietic tissue, germinal tissue, hair follicles, and the gastrointestinal lining. Despite its potential to extend survival, chemotherapy, as well as hormonal therapies, can negatively influence quality of life. Additionally, patients with metastatic cancer are more prone to experiencing declines in various aspects of quality of life due to factors such as pain, functional limitations, and emotional distress [22-24].

To the best of our knowledge, this study represents one of the initial attempts to establish a correlation between dietary patterns and the quality of life among Brazilian breast cancer patients. Given the increasing incidence of breast cancer and the potential for dietary interventions to improve quality of life, the study addresses a relevant and timely issue in cancer care [1].

However, the improvements in well-being and adherence to diets were self-reported by participants, which could introduce bias and may not accurately reflect the actual effects of the diets on quality of life. The study was retrospective in nature, which means that it relied on past data and may not have captured the dynamic nature of quality-of-life changes during the course of treatment. The sampling was voluntary, which could introduce selection bias. Therefore, larger and prospective studies are required in the future because the relationship between diet and quality of life demands more rigorous investigation.

## Conclusions

Our study reveals that restrictive diets like the ketogenic diet and intermittent fasting have limited adoption among Brazilian breast cancer patients, possibly due to their strict nature and physician concerns. Nonetheless, a significant portion of participants reported subjective improvements in well-being upon initiating these diets. High adherence correlated with better symptom management emphasizes the benefits of healthy dietary choices. Patients who adhered to their diets also showed modest physical function improvements. While our analysis did not find significant variations in other quality-of-life aspects, further investigation, especially within specific subgroups, is needed.
